# Mental health and psychosocial support interventions for individuals affected by gender-based violence in fragile contexts: A systematic review

**DOI:** 10.1017/gmh.2026.10272

**Published:** 2026-07-22

**Authors:** Tanya Ann Antony, Eamonn Whelan, Andrea Finnegan, Frédérique Vallières, Laurene Barlet, Meg Ryan, Esperanza Escorza, Geraldine Fitzgerald, Kristin Hadfield

**Affiliations:** 1 Trinity Centre for Global Health, Trinity College Dublin, Dublin, Ireland; 2 World Vision Ireland, Ireland; 3 The Library of Trinity College Dublin, Trinity College Dublin, Ireland

**Keywords:** gender-based violence, fragile contexts, psychosocial support, mental health interventions, intervention outcomes

## Abstract

Gender-based violence (GBV) is a global public health crisis exacerbated in fragile settings by gender inequalities, displacement, and resource scarcity. While mental health and psychosocial support (MHPSS) interventions aim to support people with experience of GBV, research on their effectiveness across diverse fragile contexts remains limited. In this review, we answer the following questions: (1) What type(s) of MHPSS interventions are commonly implemented with survivors of GBV in humanitarian/fragile settings? (2) What outcome(s) are targeted by MHPSS interventions implemented for those exposed to GBV in fragile settings? (3) What is the reported effectiveness of these interventions in addressing these specified outcomes? Seven electronic databases (PubMed, PsycINFO, Cochrane, CINAHL, ERIC, ProQuest, and Web of Science) and grey literature were searched for studies published up to July 2024. Following screening of 2,268 titles and abstracts, 157 full texts were screened (20% independently), with included studies analysed using narrative synthesis. Quality of studies was assessed using the Quality Assessment with Diverse Studies (QuADS). Forty-seven studies are included in this review. MHPSS interventions addressed four broad categories of outcomes: mental health, incidences of GBV, social and community outcomes, and women’s economic empowerment. MHPSS interventions for GBV survivors in fragile settings show promising mental health benefits, with the highest quality evidence found for cognitive behavioural therapy-based approaches in reducing post-traumatic stress, depression, and anxiety. Economic and psychosocial empowerment interventions also led to reduced incidences of intimate partner violence, enhanced financial autonomy, and shifts in social norms, though some interventions yielded mixed or non-significant effects. While many MHPSS interventions for people who had experienced GBV led to meaningful improvements in mental health, GBV reduction and economic empowerment, variability in effectiveness highlights the need for culturally adapted, context-specific approaches and further research on long-term effects in fragile settings.

## Impact statement

This review brings together multiple sources of evidence to advance our current understanding of the types of mental health and psychosocial support (MHPSS) interventions commonly implemented with survivors of gender-based violence (GBV) as well as which MHPSS interventions work to address psychological distress among individuals and communities who have experienced GBV within fragile contexts. Findings are of benefit to researchers and professionals involved in the planning, development, and implementation of interventions intended to improve the mental health and wellbeing of individuals affected by GBV. Specifically, this review presents current evidence for which mental health and psychosocial support (MHPSS) interventions work to address the mental health outcomes of post-traumatic stress disorder, depression, and anxiety, all of which are prevalent in fragile settings. Interventions focused on cognitive-behavioural approaches, economic interventions, and alternative therapies emerged as particularly promising. These results enhance our understanding of pathways to effectively address mental health concerns. Findings also shed light on the social- and community-level factors that maintain GBV, namely alcohol use, limited financial autonomy, and social norms. Targeting these root causes of GBV at a more ecological level can have far-reaching societal impacts, including altered social and gender norms. This review indicated limited stakeholder involvement in intervention planning, highlighting the need for future interventions to involve the community in the planning stage in order to develop culture-specific and culture-sensitive interventions. Local community involvement in implementation of interventions was found to enhance their acceptability within communities. In terms of economic impact, our findings point to the importance of involving lay people in the implementation of interventions as a cost-effective means of delivering support. Further, fostering financial autonomy of women and involvement of communities in financial programmes can contribute towards economic development in communities affected by scarcity of resources and violence.

## Introduction

Despite its pervasive occurrence across age groups and demographics, gender-based violence (GBV) remains a largely invisible human rights violation (UNICEF, 2020). A significant public health concern (Heidari and Moreno, 2016), the Inter-Agency Standing Committee (2015) describes GBV as “any harmful act that is perpetrated against a person’s will and that is based on socially ascribed (i.e. gender) differences between males and females” (p. 5). Such violence may take the form of physical, economic, psychological and sexual abuse (Ugowe, 2022). According to the UN Women Global Database on Violence Against Women (2026), approximately one in three women globally have encountered physical and/or sexual violence at least once during their lives.

The risk of experiencing GBV is particularly heightened in humanitarian and fragile contexts (Odwe et al., 2018; Lwamba et al., 2022), where gender can interact with other factors creating further inequalities and increased marginalisation among women and girls (Sweetman and Rowlands, 2016). For example, sexual violence, generally against women, is often used as a weapon of war, with significant implications for survivors (Heidari & Moreno, 2016). These include adverse mental (Rees et al., 2011), physical (Giammarioli et al., 2023) and sexual and reproductive (Mukanangana et al., 2014) consequences, as well as social consequences such as stigma and ostracization (Koos and Lindsey, 2022).

Interventions targeting individuals who have experienced GBV in fragile states have traditionally centred on empowering victim-survivors and preventing further GBV (Noble et al., 2019; Lwamba et al., 2022). However, the growing recognition of the profound impact humanitarian crises have on individuals, families and communities has shifted attention towards addressing the mental health and psychosocial support (MHPSS) needs of affected populations in these settings (Dickson and Bangpan, 2018). Existing systematic reviews have primarily focused on specific aspects of MHPSS interventions, such as psychosocial support for survivors of sexual violence in conflict and emergency contexts (Andersen and Buttigieg, 2024), or on the implementation of MHPSS for women and children in fragile contexts (Kamali et al., 2020). Additionally, some reviews have examined the effectiveness of MHPSS interventions to improve mental health outcomes among survivors of GBV in such settings (e.g., see Tol et al., 2013; Lakin et al., 2022; Keith et al., 2023). However, these reviews predominantly focus on mental health and well-being outcomes or the reduction in rates of GBV. As a result, we have a limited understanding of the full scope of MHPSS interventions commonly implemented for people in fragile contexts exposed to GBV. Specifically, there is limited insight into the diverse outcomes these interventions aim to achieve, beyond mental health or GBV reduction, and how effectively these interventions meet the varied needs of individuals exposed to GBV in such contexts.

To address these gaps, this review sought to comprehensively assess the range of MHPSS interventions for GBV survivors in fragile contexts, the broader psychological, social and economic outcomes they target and their overall effectiveness in improving these outcomes. Specifically, we ask:

1. What type(s) of MHPSS interventions are commonly implemented with survivors of GBV in humanitarian/fragile settings?
2. What outcome(s) are targeted by MHPSS interventions implemented for those exposed to GBV in fragile settings?
3. What is the reported effectiveness of these interventions in addressing these specified outcomes?

## Methods

The systematic review followed the Preferred Reporting Items for Systematic reviews and Meta-Analyses (PRISMA) guidelines (Page et al., 2021) and was pre-registered on Prospero (PROSPERO 2024 CRD42024560281).

The Population, Intervention, Comparison and Outcome (PICO) model was used to identify key components to be considered when reviewing eligibility of existing literature (Richardson et al., 1995; Table 1). The review included empirical qualitative, quantitative or mixed methods studies that meet the inclusion criteria (see Table 1).

**Table 1.** PICO framework Table 1. long description.

PICO framework		
	Inclusion criteria	Exclusion criteria
Population	People who have experienced or are experiencing acts of GBV, without regard to gender, race or ethnicity. This includes not only those with direct experience of GBV but also people, families or communities that frequently witness acts of GBV. For example, a child in a household who witnesses a parent perpetuating GBV against their spouse, or a population hosting internally displaced people in a fragile setting where acts of GBV are perpetrated.	People who have not directly or indirectly experienced acts of gender-based violence.
Intervention	MHPSS interventions delivered to people, families or communities who have directly experienced or witnessed acts of GBV within a fragile setting. MHPSS interventions refer to any type of local or outside support that intends to protect or promote psychosocial well-being and/or prevent or treat mental health conditions.	MHPSS interventions delivered to people who have not endured acts of gender-based violence.
Comparison	No comparison used.	No comparison used.
Outcome	Any outcome among those who have experienced GBV resulting from participation in the MHPSS intervention (i.e., mental health, resilience, women’s empowerment, GBV, etc.).	Any outcomes that are not associated with the delivery of an MHPSS intervention.

### Search strategy

Assisted by a University subject librarian (GF), we searched for research publications across the following electronic databases and websites: PubMed, PsycINFO, Cochrane, CINAHL, ERIC, ProQuest, Web of Science and Google Scholar; The WHO library catalogue; the United Nations digital library; The Mental Health and Psychosocial Support Network; the Red Cross and Red Crescent Reference Centre for Psychosocial Support; World Vision International; Relief Web; European Union External Action; and leading GBV non-governmental organisation (NGO) websites. We further searched the grey literature in line with the Cochrane Handbook for Systematic Reviews of Interventions (Higgins et al., 2019) to manually identify relevant policy documents, guidelines, dissertations and reports. We also manually reviewed the reference lists of all included studies.

The search strategy included terms associated with MHPSS interventions, fragile states and GBV. Full search terms are available in our review protocol (PROSPERO 2024 CRD42024560281). This review follows the Inter-agency Standing Committee’s (2007) definition of MHPSS, which is “any type of local or outside support that means to protect or promote psychosocial well-being and/or prevent or treat mental health conditions.” We use UNICEF’s (2018) definition of fragile settings as “contexts where there is an accumulation and combination of risks as a result of context-specific underlying causes combined with insufficient coping capacity of the state, system and/or communities to manage, absorb or mitigate those risks.” All searches were conducted between 21 June and 24 July 2024. No date restrictions on year of publication were applied. Only English language articles were included.

### Data selection and extraction

**Figure 1.**
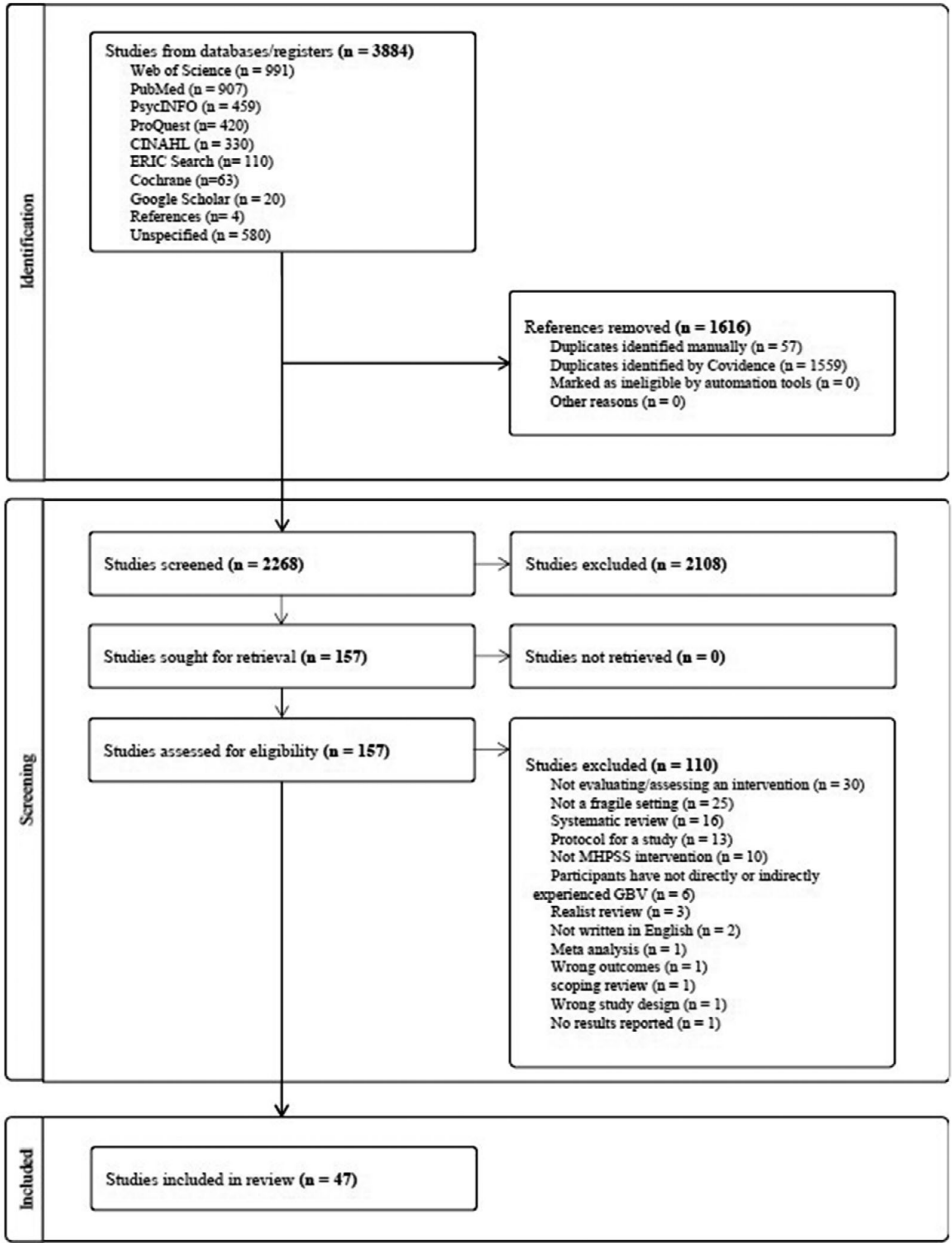
PRISMA flow diagram. Figure 1. long description.

Articles were transferred to Covidence for data management. Author EW independently screened all titles and abstracts to identify articles suitable for full-text screening, all of which were reviewed and assessed against the inclusion and exclusion criteria (Figure 1). In line with our pre-registration and to minimise bias and maximise rigour, KH independently reviewed a random 20% of all titles and abstracts, and a combination of KH, FV and MR independently reviewed a random 20% of the full texts. We then extracted the following data from the selected studies: authors and year of publication, study setting, design, objectives, sampling methods, sample size, sample demographics, form of GBV exposure, MHPSS intervention delivered, research outcomes of interest, outcome assessment measures and intervention results.

### Data synthesis

As the review was based on heterogeneous studies that adopted diverse methodologies, we employed an outcome-based narrative synthesis approach to integrate the findings across studies (Higgins et al., 2019). We use the term effectiveness to refer to whether interventions produced their intended outcomes under the real-world conditions of fragile and humanitarian settings (including delivery by non-specialists and lay providers, heterogeneous and often displaced populations and variable implementation contexts), rather than efficacy demonstrated under the controlled conditions of explanatory trials. We recognise that efficacy and effectiveness lie on a continuum, and that a minority of included studies used more controlled designs; the majority, however, were conducted under pragmatic field conditions. As the research questions focused on the outcomes of MHPSS interventions, the extracted data were categorised based on study outcomes. Both primary and secondary outcomes were considered in the analysis, and as most studies had multiple outcomes, a single intervention could be attributed to multiple categories (e.g., both mental health outcomes and incidences of GBV). Patterns across studies within each category were explored and then grouped based on similarities.

### Quality appraisal

We assessed the quality of included studies using the Quality Assessment with Diverse Studies (QuADS) tool (Harrison et al., 2021), an instrument to appraise the quality of research methods, evidence and reporting. QuADS assesses studies along 13 criteria. These include theoretical underpinning of the research, statement of research aims, description of research setting and population, appropriateness of study design, provision of recruitment data, justification of method of analysis, appropriateness of analytical method, evidence of stakeholder consideration and discussion of study strengths and limitations. The studies included in this review were assigned a score between 0 (indicating lacking in that criterion) and 3 (indicating meeting the criterion) for each domain, for a maximum total score of 39. While all studies meeting the inclusion/exclusion criteria were included in this review regardless of their QuADS score, studies assessed as being of lower methodological quality were given less weight in the synthesis and interpretation of our findings. Considering the potential impact of study quality on the robustness of the evidence presented, our conclusions were primarily driven by studies demonstrating more rigorous design and implementation.

## Results

Database searches identified 3,884 studies. Following the removal of duplicates, 2,268 articles were screened. A total of 157 articles were selected for full-text screening, of which 110 articles were excluded through the full-text review. The remaining 47 articles were included in the review (Figure 1).

### Description of selected studies

#### Location and setting of interventions

**Figure 2.**
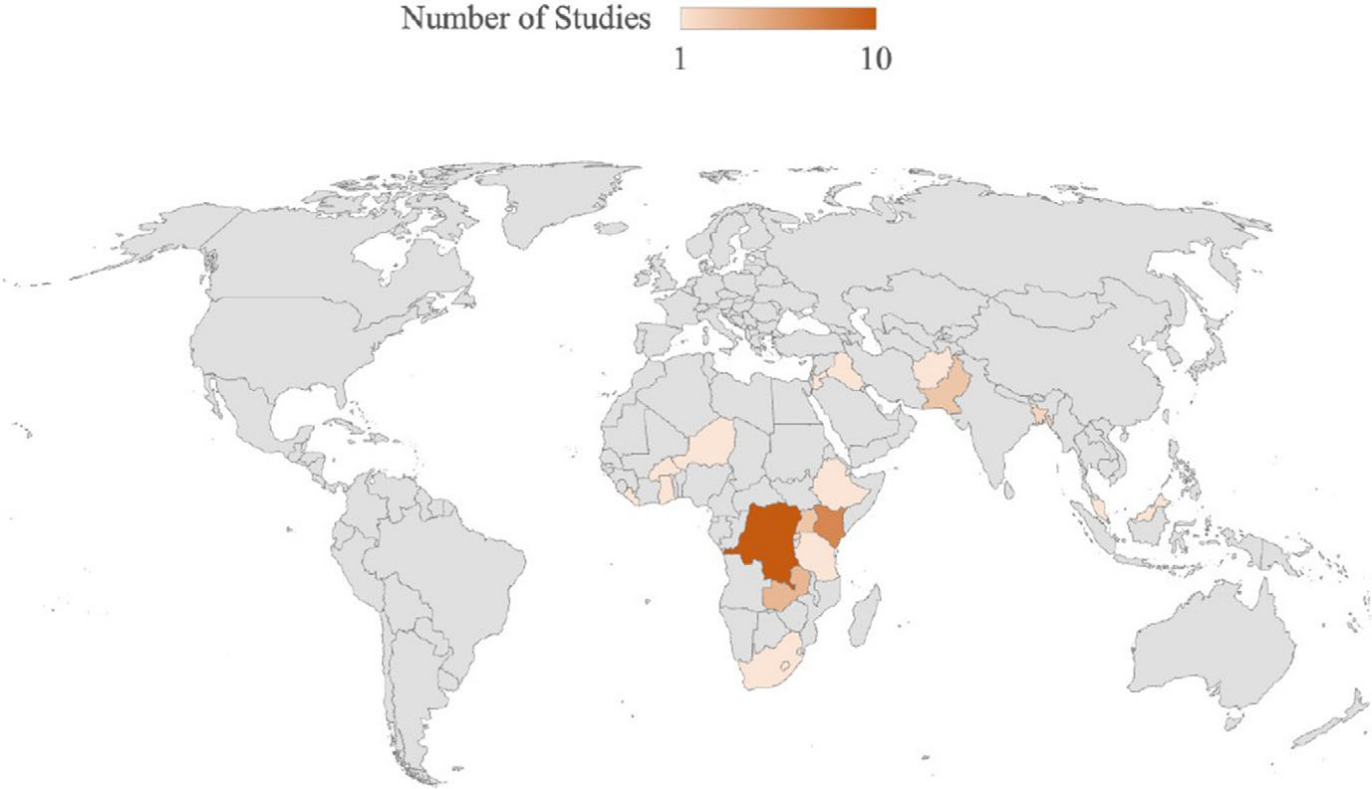
Map of study locations. Figure 2. long description.

Despite no date restrictions, only studies published between 2010 and 2024 fit our criteria. The country accounting for the most studies was the Democratic Republic of Congo (DRC; *n* = 10), followed by Kenya (*n* = 7), Lebanon (*n* = 4) and Zambia (*n* = 4). The remaining studies were conducted in Afghanistan, Bangladesh, Burkina Faso, Ethiopia, Ghana, Iraq, Jordan, Lesotho, Liberia, Malaysia, Nairobi, Niger, Pakistan, Palestine, Rwanda, South Africa, Syria, Tanzania and Uganda (Figure 2). Studies were conducted in villages and rural settings (*n* = 14), in refugee camps or settlements (*n* = 11), in urban settings (*n* = 8), in peri-urban slums and communities (*n* = 5), in shelters or safe houses for survivors of GBV (*n* = 4), in health facilities (*n* = 2) and in other settings that are mixed or not fully specified in the papers (*n* = 3). See Supplementary File 1 for a summary of the study characteristics of each included study.

#### Sampling and demographics

Most studies used purposive (*n* = 23) or convenience sampling (*n* = 11), often recruiting participants through local partner organisations, schools, NGOs, door-to-door community outreach, refugee settlements, HIV clinics, community care groups or clients of mental health and domestic violence services. Cluster sampling was employed in four studies for recruitment and assignment to intervention or control groups, while three studies used simple or stratified random sampling methods. Finally, one study used voluntary sampling via radio announcements. It was not possible to identify the sampling strategy for five of the studies.

The sample size in the qualitative studies included in the review ranged from 2 to 469 participants, with an average of 101 participants. The average sample size in the quantitative studies was 592, ranging from 11 to 4,289 participants. Across all interventions, participants’ ages ranged from 11 to 69 years.

The interventions identified in this review targeted a wide range of groups affected by GBV. These included women who had experienced or witnessed various kinds of GBV (*n* = 27), adolescents and young adults who experienced or were exposed to GBV (*n* = 12), couples and families among whom intimate partner violence (IPV) was prevalent (*n* = 6), men who have perpetrated IPV (*n* = 1) and a mixture of different groups (*n* = 1). Interventions aimed to foster change at the individual level (*n* = 28), community level (*n* = 10), family level (*n* = 7) and school level (*n* = 1). Several forms of GBV were addressed across the interventions. Most of the studies focused on IPV (*n* = 17) and direct or indirect exposure to sexual violence (*n* = 13). Conflict- or war-related violence, such as the use of violence against women as a weapon of war; psychological/emotional abuse; parental IPV; domestic violence; verbal violence; economic violence; human trafficking; child marriage and forms of violence not otherwise specified were also targeted by these interventions.

#### Implementation of interventions

Interventions were implemented by psychosocial assistants (*n* = 4), therapists or counsellors (*n* = 4), community health workers/volunteers (*n* = 4), social workers (*n* = 3), research assistants (*n* = 2), psychologists (*n* = 2), trained mentors or teachers (*n* = 2), case workers (*n* = 2) and field agents (*n* = 1). Lay community members (*n* = 12), including refugees working for women’s empowerment, lay counsellors and local women under regular supervision, were often trained to implement the intervention. Alternative therapy interventions were delivered by specialists, including a music producer and art and yoga instructors. Few studies were careful to consider cultural norms around interactions between genders by matching facilitators to group members of the same gender, such that male groups had male facilitators and female groups had female facilitators.

### Outcomes and effectiveness of interventions

Included studies were grouped based on four broad categories of outcomes: *mental health outcomes* (*n* = 24), most commonly depression, PTSD, anxiety, psychological distress and functional impairment; *incidences of GBV* (*n* = 18), typically operationalised through self-reported experiences of IPV, domestic violence, sexual assault, child marriage or harsh discipline; *social- and community-level outcomes* (*n* = 7), including felt stigma, social capital, acceptability of violence, couple communication, help-seeking behaviours and community cohesion; and *women’s economic empowerment* outcomes (*n* = 5), including financial autonomy, decision-making power, engagement in income-generating activities, food security and employment status. Given the diversity of measurement tools and reporting formats across studies, it was not feasible to meaningfully summarise baseline prevalence rates across included studies.

#### Mental health outcomes

Most common mental health outcomes were depression (*n* = 14), post-traumatic stress disorder (PTSD; *n* = 9) and anxiety (*n* = 7). Nine of the included studies targeting mental health outcomes were rooted in the theory of cognitive behavioural therapy (CBT) and thus based on the idea that emotions and behaviours are shaped by people’s perceptions of events, rather than the event itself (Beck, 1964; 2011). CBT, as a structured form of psychotherapy, therefore aims to alleviate psychological distress through the identification and subsequent alteration of negative thought patterns and behaviours that may arise following a distressing event (Hofmann et al., 2012). O’Callaghan et al. (2013), for example, implemented a 15-session manualised treatment of a culturally modified trauma-focused cognitive behavioural therapy (TF-CBT) intervention among adolescent girls in the DRC, who had direct or indirect experience of rape or sexual abuse. In this single-blind randomised control trial, the TF-CBT group showed significant reductions in trauma symptoms, depression and anxiety, as well as behavioural outcomes including conduct problems and pro-social behaviour. These changes persisted to the 3-month follow-up. A feasibility study by Latif et al. (2021) in Pakistan (also adopting culturally adapted TF-CBT) found TF-CBT reduced symptoms of PTSD, depression, anxiety and disability; furthermore, the intervention received positive informal feedback from participants.

Three studies investigated the effectiveness of cognitive processing therapy (CPT), a form of CBT targeting psychological distress. In the DRC, Bass et al. (2013) found that women in the CPT intervention group showed reductions in symptoms of depression, anxiety and PTSD at the end of treatment and at the 6-month follow-up. This effect was maintained in more than half of the sample at a 2-year follow-up (Bass et al., 2022). Greene et al. (2021) similarly implemented an advocacy and CPT intervention group in a refugee camp in Tanzania, which was found to be effective among female survivors of IPV in reducing psychological distress.

Problem Management Plus (PM+) – a transdiagnostic, low-intensity, CBT-based intervention, developed by the World Health Organization (WHO), and delivered by trained laypeople – was implemented in two projects in Kenya (Dawson et al., 2016; Bryant et al., 2017). The programme includes behavioural activation, problem-solving and stress reduction strategies, as well as means of accessing social support. Results from these studies indicated that PM+ led to reductions in psychological distress and PTSD scores, with a dose–response effect between the number of sessions attended and psychological distress.

Alternative therapies showed promising outcomes such as reductions in depression, anxiety and PTSD symptoms; enhanced feelings of joy, pride and hopefulness; and increased self-esteem, improved self-reports of physical and emotional health, as well as improved general health status, among adolescent girls and women. These interventions included a music therapy intervention *Healing in Harmony* (Cikuru et al., 2021), a yoga intervention *Healing and Resilience after Trauma* (HaRT) (Namy et al. 2022) and an art-based intervention involving training in creating art, making art through different mediums, sharing the meaning of participant’s artwork within the group and an opportunity to showcase their work at an exhibition (Abdulah and Abdulla, 2019). Other interventions targeting mental health outcomes involved narrative exposure therapy with female former child soldiers (Robjant et al., 2019), interpersonal psychotherapy among HIV-positive women (Meffert et al., 2021) and family therapy based on solutions-focused and systems-based approaches, for families with dysfunctional interactions (Puffer et al., 2020), yielding effects ranging from non-significant to moderate on mental health outcomes.

Several interventions worked to build empowerment and personal agency of women, but also included mental health outcomes. Economic and psychosocial empowerment interventions, involving skills building (Hirani et al., 2016), as well as psychoeducation and identification of safety measures to protect against GBV (Mutisya et al., 2018), showed positive effects on mental health, specifically reductions in psychological distress. Conversely, Kalra et al.’ (2024) personal agency training intervention resulted in higher rates of probable anxiety, depression and PTSD in the intervention group, compared to the control group. However, baseline prevalence of these conditions was low (~5%), likely reflecting the protracted nature of the settlement and participants’ prior access to mental health services. While the authors suggest that increased introspection and revisiting of trauma during the intervention may have contributed, other factors may also be relevant, including concurrent policy changes within the camp and the relatively short follow-up period of 6 months.

While most interventions resulted in positive effects for mental health outcomes, in some cases, no significant effect on mental health was observed (Park, 2022; Friedberg et al., 2023). For Friedberg and colleagues, this could be because the mental health data collection was at endpoint, after significant attrition. In Park, one possibility posited for why the intervention did not impact psychological wellbeing but did impact the other outcomes is that the endpoint is 12 months after the intervention implementation, by which point they suggest that positive effects of the intervention on psychological well-being may have been reduced.

#### Incidences of GBV

After mental health outcomes, incidence of GBV (*n* = 18) was the second most reported outcome used to assess the effectiveness of MHPSS interventions among this population. Incidences of GBV were commonly assessed through self-report measures completed by individuals affected by GBV.

Common Elements Treatment Approach (CETA), a cognitive-behavioural transdiagnostic intervention, was implemented among couples in Zambia (Murray et al., 2020; Kane et al., 2021) by trained local lay counsellors. Authors note immediate, significant treatment effects, with CETA leading to reductions in physical/sexual violence, threatened violence and male partner alcohol use. Similar effects were maintained at the 12- and 24-month follow-ups. Murray et al.’ (2021) qualitative exploration into the mechanisms of change revealed that participants found the CETA safety strategies for managing conflict particularly helpful. Awareness of the connection between perpetrator’s alcohol use, IPV perpetration and inability to meet family responsibilities facilitated reduced alcohol consumption, which fostered trust and improved relationship quality.

Evaluations of a solution-focused family therapy pilot intervention (Puffer et al., 2020) and a family intervention for HIV-affected families (Chaudhury et al., 2016) led to reduced alcohol use by the perpetrator and reduced alcohol-related conflict, improved relationship quality and reductions in IPV. Participants reported improved communication and conflict resolution, less money spent on alcohol, clearer roles and responsibilities and increased decision-making power for women. These changes also contributed towards improvements in child and caregiver mental health outcomes.

Economic and psychosocial empowerment interventions aimed at helping survivors of IPV become self-sufficient also resulted in lower incidences of emotional and physical IPV. The intervention components included psychosocial empowerment through group counselling and CBT, vocational skills, business training (Park, 2022) and economic strengthening combined with gender sensitive family coaching (Ismayilova et al., 2018).

Among adolescent girls, interventions focused on reducing sexual violence, GBV in brother–sister sibling dyads and child marriage. Sarnquist et al. (2014) implemented an empowerment and self-defence training intervention in Kenya, which yielded significant reductions in the annual rate of sexual assault and increased disclosures of sexual abuse. Participants also reported using self-defence skills against attempted sexual harassment. Other community interventions – vocational training in Gaza (Thabet et al. 2011), psychosocial components such as provision of information about GBV in Kenya (Mutisya et al., 2018) and advocacy groups in Tanzania (Greene et al., 2021) – contributed towards lower incidences of IPV and domestic violence.

#### Social and community level outcomes

Social norms surrounding GBV and gender that maintain and perpetuate violence against girls and women were addressed in seven studies. Addressing social norms around GBV resulted in improved quality of sibling relationships marked by greater emotional connection and reduced gendered division of tasks in Jordan and Niger (Koris et al., 2023), and slightly lower rates of child marriage in Bangladesh (Naved et al., 2024). The topics covered included social expectations and restrictive norms, as well as discussions on gender, power and violence.

In an intervention targeting the reformation of social norms and practices among men that increase the risk of GBV among Syrian refugees in Lebanon, Veale et al. (2020) implemented a community project with sessions focused on promoting peaceful interactions, reducing GBV, child protection and caregiving and greater community harmony. In qualitative interviews, the male participants identified the usefulness of discussing the implications of their role as breadwinner, and the psychosocial support and well-being resulting from group membership was valued.

In a qualitative study, Mannella and colleagues (2018) explored how narrative storytelling can help reduce distress caused by GBV among women in Afghanistan. Women discussed the shame, stigma and fear of blame associated with GBV. However, they also reported that storytelling in a supportive environment offered relief from suffering.

The impact of MHPSS interventions on the acceptability of GBV and related social norms was also investigated. An economic and psychosocial empowerment intervention (Park, 2022) and a social norms-based workshop and poster campaign (James et al., 2021) resulted in a significant reduction in acceptability and justification of GBV. A life skills intervention for adolescents (Asghar et al., 2018) resulted in increased belief in girls being deserving of the same opportunities as their male counterparts and increased perceived social support. In a similar vein, four interventions also showed improvements in support seeking (Hall et al., 2014; James et al., 2021) and disclosures of abuse (Sarnquist et al., 2014; Koris et al., 2023). These included a CPT intervention, a family-based intervention and interventions addressing women’s empowerment and prevailing social norms.

Bass et al. (2016) implemented a Village Savings and Loans Associations intervention among adult female survivors or witnesses of sexual violence in villages in the DRC. Apart from the resultant economic benefits of the intervention, the women in the intervention group reported significant reductions in their experience of stigma about experiencing GBV. Similar reductions in felt stigma were observed in a CPT (Murray et al., 2018) among women in rural DRC, which was maintained at a 6-month follow-up.

#### Women’s economic empowerment

Women’s economic interventions (*n =* 5) focused on economic empowerment on the premise that greater financial autonomy would result in reduced IPV. The economic outcomes investigated in these interventions include economic livelihoods (Park, 2022); women’s engagement in the labour market, household assets and food intake (Bass et al., 2016); women’s empowerment, decision-making power and financial autonomy (Ismayilova et al., 2018); self-reported employment status (Hirani et al., 2016); and participation in income-generating activities, skills development, food insecurity and social agency (Kalra et al., 2024).

An intensive long-term intervention, Park’s (2022) *Women Training and Integration* model, included group and individual counselling sessions, vocational skills and business training, educational components, as well as healthcare and childcare services. Kalra et al. (2024) implemented a 2-day personal agency training, which comprised self-reflection, recognition of personal aspirations and identifying strategies of action. The intervention also involved a component for male partners to pre-emptively encounter possible feelings of emasculation that may stem from their female partner’s economic empowerment. Other programmes focused on economic strengthening (Ismayilova et al., 2018) and community loan associations (Bass et al., 2016). These interventions resulted in increased financial autonomy, food consumption, engagement in income-generating activities, expenditure, skill building and decision-making power among women.

### Quality appraisal

The QuADS scoring revealed that the strengths in the included studies were a clear description of the target population and use of an appropriate study design to address the established aims. While the methods of analysis in most studies were deemed appropriate, many studies did not provide sufficient justification for their analysis. A weakness across the studies was the lack of stakeholder involvement in the research design.

## Discussion

The purpose of this review was to explore the types of MHPSS interventions commonly implemented with survivors of GBV in humanitarian contexts, to identify the outcomes targeted by these interventions, and to examine the reported effectiveness of these interventions in targeting the specified outcomes. We identified 47 studies that fit the inclusion criteria for this review, with included interventions conducted across 22 countries. MHPSS interventions typically addressed four broad categories of outcomes: mental health-related outcomes, incidences of GBV, social- and community-level outcomes and women’s economic empowerment. In this Discussion, we synthesise our findings thematically and relate each theme to these three questions: the types of interventions implemented (RQ1), the outcomes they targeted (RQ2) and their effectiveness in addressing those outcomes (RQ3).

In terms of the types of interventions implemented and their effectiveness, transdiagnostic and trauma-informed CBT has a strong evidence base for long-term change in mental health outcomes among survivors of GBV in conflict-affected settings (e.g., see St John and Walmsley, 2021; Lakin et al., 2022; Cai et al., 2025), and our review reinforces this, with CBT-based interventions showing consistent positive effects on mental health outcomes across multiple studies, populations and settings (e.g., see Bass et al., 2013; O’Callaghan et al., 2013; Bryant et al., 2017; Murray et al., 2020). We further found evidence for CBT for couples as a way to reduce rates of GBV, with interventions involving self-defence training for adolescents, group counselling for women, economic and social empowerment-focused interventions for women and family interventions having similar outcomes for incidences of GBV. Beyond CBT, preliminary evidence from this review suggests that alternative therapies (including art, music and yoga) may offer benefits for psychological well-being among GBV survivors, though each has been evaluated in only a single study and these findings are in need of replication. Similarly, while the small number of economic empowerment interventions showed promising effects on financial autonomy and, in some cases, reductions in IPV, the limited number of studies precludes strong conclusions about the effectiveness of these approaches as a category.

The evidence base identified in this review overwhelmingly focused on women and girls, with only one study targeting men and none involving gender-diverse individuals. In terms of the nature of interventions, group-based interventions were a means of reducing felt stigma and enhancing perceived support, as experienced by survivors of GBV. Among men with a history of GBV perpetration, group discussions fostered self-reflection and a sense of community. Humanitarian aid in fragile contexts often focuses on women and children, leaving men feeling forgotten (e.g., see Griffiths, 2015; Veale et al., 2020; Alexandre et al., 2025); this may impact how interventions on GBV are received, influencing their efficacy. Inclusion of men, in the form of including men and boys to challenge prevailing attitudes, for example, may be one way to promote greater acceptability and effectiveness of interventions seeking to address issues of GBV in fragile contexts. Indeed, Barker et al.’ (2010) systematic review found that discussions on the meanings of masculinity and the role of men were effective in changing behaviours and fostering equitable relationships between men and women. Our findings resonate with the WHO and UN Women RESPECT framework for preventing violence against women (WHO, 2019, 2025), particularly the “R” (Relationship skills strengthened) strategy, which emphasises improving communication, conflict management and shared decision-making among couples and families an approach reflected in several interventions in this review that engaged men alongside women (e.g., see Murray et al., 2020; Veale et al., 2020; Kane et al., 2021; Falb et al., 2023). A recent update to the RESPECT evidence base found that relationship skills strengthening remains one of the least-evidenced strategies, particularly in humanitarian settings (Ullman et al., 2025), underscoring the need for further research in this area.

Few interventions included in this review involved stakeholders in the planning stage: a notable gap given the importance of eliciting change from within social systems in fragile contexts (De Weijer, 2012), and the need to understand community expectations when proposing developments in conflict-affected settings (Singh et al., 2021). The interventions that did involve stakeholders did so in the form of partnering with local organisations; conducting focus groups and key informant interviews with the intervention target group, healthcare workers, other service providers in the region and community members to aid planning; or adaptation of pilot interventions following formal or informal feedback. Where included, the involvement of lay people in the implementation of MHPSS interventions was widely accepted in the target populations, similar to what has been found in other intervention research in fragile contexts (Ryan et al., 2021). This may be attributed to the layperson’s understanding of the cultural context of the intended population, as well as participant familiarity with the lay community members, and vice versa. Including laypersons in intervention implementation may be a feasible and cost-effective means of implementation, which also equips facilitators with skills that may be beneficial for the community (Greene et al., 2022). Cultural sensitivity is also relevant to the evaluation of interventions. While some studies employed culturally adapted versions of standardised measures or conducted local validation (e.g., see Bass et al., 2013; Latif et al., 2021), many applied instruments developed in high-income Western contexts without reporting on the processes or translation or whether these measures were validated in the population being studied.

Turning to the outcomes targeted, the range of measured outcomes was itself uneven. Given the well-documented implications of GBV for self-esteem, particularly in the additional context of state fragility where displacement, loss of social networks and economic dependency may further compound survivors’ sense of self-worth (e.g., see Mbaluka, 2018), the limited attention to self-esteem as a measured outcome is a notable gap. A small number of studies reported improvements in self-esteem, primarily those involving alternative therapies such as art and music (e.g., see Abdulah and Abdulla, 2019). Economic empowerment approaches may also hold promise in this regard, given their potential to increase agency, financial autonomy, and reduced dependence on male counterparts (Hirani et al., 2016; Ditter and Qi, 2024), but further research incorporating validated and culturally appropriate measures of self-esteem is needed to better understand this dimension of recovery among GBV survivors in fragile settings.

Outcomes of economic interventions that aim to enhance women’s financial autonomy included greater decision-making power, skills building, and engagement in income-generating activities, which consequently reduce rates and acceptability of GBV. It has been suggested that women’s economic empowerment may increase GBV if men feel their traditional provider role is threatened (Tappis et al. 2016). However, none of the economic interventions in this review documented such an increase. Ismayilova et al. (2018) found significant reductions in emotional violence, with larger effects in the arm that combined economic strengthening with family coaching involving husbands. Park (2022) reported substantial reductions in both emotional and physical IPV following an intensive programme combining vocational training with psychosocial therapy. Kalra et al. (2024) included a component for male partners specifically designed to pre-empt feelings of emasculation stemming from women’s economic empowerment. These findings suggest that the risk of backlash may be mitigated when economic interventions are designed with attention to household gender dynamics and, where possible, actively involve men.

Interventions targeting GBV often aimed to address factors thought to perpetuate GBV, including reduced stigma, increased support-seeking behaviours and disclosures of abuse. These factors are consistent with previous studies citing male alcohol use (e.g., see Mootz et al., 2018; Gilchrist et al., 2023), women’s lack of financial autonomy (e.g., see McDougal et al., 2019) and the existing social beliefs and norms regarding gender and power (e.g., see Raftery et al., 2022) as underpinning GBV perpetuation/victimisation. Awareness of the impact of alcohol use on violence in the home and learning concrete skills to reduce alcohol intake were found to be useful mechanisms to reduce GBV – and specifically IPV – perpetration. Targeting children, adolescents and women through workshops; holding poster campaigns; having group discussions; conducting life skills training; or implementing empowerment interventions were also found to lower rates of child marriage and increase disclosures of abuse.

Several contextual factors appear to shape intervention effectiveness and feasibility. Notably, the settings represented in this review differ in ways that have direct implications for both the nature of GBV and the feasibility of intervention delivery. In active conflict settings, GBV often takes the form of conflict-related sexual violence (e.g., see Bass et al., 2013; Falb et al., 2023) and ongoing insecurity disrupts intervention delivery and population displacement creates challenges for retention. Bass et al. (2013), for example, reported major security incidents in all study villages during the trial. In contrast, protracted refugee and displacement settings, such as those in Tanzania (Greene et al., 2021) and Rwanda (Kalra et al., 2024), offer greater stability for sustained intervention delivery but present different drivers of GBV and distress. In protracted displacement, ongoing structural stressors such as restrictions on movement, employment and documentation are more consistent predictors of mental health difficulties and negative family dynamics than past war exposure (Miller and Rasmussen, 2017; Khraisha et al., 2026), with economic stress, shifts in gender roles and harmful alcohol use also playing important roles (Mootz et al., 2018; Gilchrist et al., 2023). The IASC guidelines (Inter-Agency Standing Committee, 2007) recognise these distinctions, recommending that structured psychological interventions be introduced once basic safety and community supports are established, rather than during acute emergency phases. The interventions with the strongest evidence base in this review, such as CPT and CETA, required sustained delivery over multiple sessions, which may be more feasible in protracted or stable settings.

Several cross-cutting patterns emerged across the interventions in this review. *First*, interventions delivered by trained non-specialists – including psychosocial assistants (Bass et al., 2013), community social workers without clinical qualifications (O’Callaghan et al., 2013), trained lay providers (Murray et al., 2020) and local women with advocacy experience (Sarnquist et al., 2014) – produced outcomes comparable to or exceeding those reported in trials using professional facilitators, consistent with broader evidence on task-shifting in humanitarian settings (e.g., see Ryan et al., 2021). *Second*, intervention intensity and duration varied considerably, from Sarnquist et al.’s (2014) six-session empowerment programme to Park’s (2022) year-long multifaceted intervention, yet longer or more intensive interventions did not uniformly produce stronger effects; rather, the alignment between intervention content, target population and the specific outcome measured appeared to be most important. *Third*, in relation to the effectiveness of GBV-reduction interventions specifically, the engagement of men emerged as a potential factor associated with impact: the few interventions that actively engaged men, whether through couples-based programming (Murray et al., 2020; Falb et al., 2023) or family-level coaching involving husbands and extended family (Ismayilova et al., 2018), were among those showing the most notable reductions in IPV, suggesting that gender inclusion may be particularly important when violence reduction is a primary outcome. However, the heterogeneity of intervention designs, populations and outcome measures across the included studies, together with the absence of head-to-head trials, limits the extent to which systematic comparisons across intervention characteristics can be drawn.

While this review identified promising outcomes across multiple intervention modalities (CBT-based therapies, economic empowerment programmes, alternative therapies and community-based approaches), where there was a comparator group, in almost all cases the comparator was a control or waitlist condition, rather than a comparison of different active interventions against one another. This means that although we can identify interventions that show positive effects relative to no treatment, we cannot determine which approaches produce the strongest or most sustained impacts for particular outcomes or populations. Future research should prioritise head-to-head trials comparing different intervention types, as well as dismantling studies that identify which specific components of multi-component programmes drive change. Without such evidence, practitioners in resource-constrained fragile settings are left without clear guidance on how to allocate limited resources to the interventions most likely to achieve their desired outcomes.

While we used a rigorous search strategy incorporating a robust search of academic databases as well as the grey literature, this review is not without limitations. *First*, as the included studies were heterogeneous in methodology and outcomes, we could not conduct a meta-analysis. For example, depression was variously assessed using the PHQ, HSCL, HADS and locally developed instruments. The considerable variation in measurement tools, scoring conventions and reporting formats across included studies limits the comparability of findings and highlights a need for greater standardisation in outcome measurement in this area. *Second*, we aimed to include qualitative studies in this review, as they can provide insight into mechanisms of change, participant experiences and contextual factors that quantitative measures of efficacy alone cannot capture. However, the relatively small number of qualitative studies available in this area limited the depth of qualitative synthesis possible, highlighting a need for further primary qualitative research on participants’ experiences of MHPSS interventions in fragile settings. *Third*, despite efforts to mitigate publication bias through the inclusion of grey literature, the findings may overrepresent interventions with positive outcomes. *Finally*, the restriction to English language studies may have excluded relevant research from non-Anglophone fragile settings.

## Conclusion

The current review offers valuable insights for the development of future interventions. The findings are based on data collected across multiple studies, synthesised to provide researchers and professionals with a starting point for future MHPSS interventions for GBV. Findings suggest that MHPSS therapeutic interventions for this population are typically based in cognitive-behavioural theory, with alternative therapies, couples’ and family therapy, group counselling and discussions and community-based economic and psychosocial empowerment interventions also being common types of interventions. The second major finding is that four broad domains, specifically mental health outcomes; incidences of GBV; social outcomes, reflected in changes in attitudes and perception of stigma; and economic empowerment, are the most targeted outcomes in such interventions. Lastly, in terms of the effectiveness of interventions, CBT interventions resulted in long-term reduction of depression, anxiety and PTSD symptoms, as well as reduced incidences of GBV. Interventions targeting underlying maintaining factors of violence can be especially effective in reducing incidences of GBV, with altering social norms and practices being vital in such a breakthrough.

We found that interventions grounded in CBT show wide applicability and yield desired results, specifically for mental health outcomes. We recommend group-based interventions to reduce stigma, build resilience and enhance support among survivors, while alternative therapies can also foster well-being. We recommend that community-based programmes in humanitarian settings take the form of workshops, poster campaigns, group discussions and life skills training for those affected by GBV, to reframe societal norms and stigma around gender, power and violence. Economic empowerment programmes for women are also recommended to increase self-sufficiency, self-efficacy and financial autonomy.

We encourage the implementation of MHPSS interventions among men in fragile settings, including displaced populations where men assume the primary role of “breadwinner” and “protector” in a new environment. This will help meet their unmet needs for community support, facilitate stress management and foster self-reflection. Stakeholder involvement is strongly recommended in the planning of interventions to offer insight into aspects unique to specific cultures and fragile states and facilitate the tailored development of interventions. Involving local community members following appropriate training can enhance understanding of local needs and public acceptance of interventions.

## Supporting information

10.1017/gmh.2026.10272.sm001 Antony et al. supplementary material Antony et al. supplementary material

## Data Availability

The current review is based on published research, all of which is cited. The PRISMA flow diagram (Figure 1) described the screening process and Supplementary File 1 offers a summary of study characteristics. Further details are available from the corresponding author on reasonable request.

## Supplementary material

The supplementary material for this article can be found at http://doi.org/10.1017/gmh.2026.10272.

## Long descriptions

### Table 1. Long description

The table is titled P I C O framework and consists of three columns: P I C O category, Inclusion criteria, and Exclusion criteria.

* Population: Inclusion includes people who have experienced or witnessed acts of G B V, regardless of gender, race, or ethnicity, including families or communities in fragile settings. Exclusion includes people who have not directly or indirectly experienced acts of gender-based violence.

* Intervention: Inclusion includes M H P S S interventions delivered to those who experienced or witnessed G B V in fragile settings to promote psychosocial well-being or treat mental health conditions. Exclusion includes M H P S S interventions delivered to people who have not endured acts of gender-based violence.

* Comparison: Both inclusion and exclusion criteria state No comparison used.

* Outcome: Inclusion includes any outcome resulting from the M H P S S intervention, such as mental health, resilience, women’s empowerment, or G B V. Exclusion includes any outcomes not associated with the delivery of an M H P S S intervention.

Navigate back to Table 1..

### Figure 1. Long description

The flowchart is divided into three vertical phases: Identification, Screening, and Included.

1. Identification Phase:

- Top box: Studies from databases/registers (n = 3884). Sources include Web of Science (n = 991), PubMed (n = 907), Psyc I N F O (n = 459), ProQuest (n = 420), C I N A H L (n = 330), E R I C Search (n = 110), Cochrane (n = 63), Google Scholar (n = 20), References (n = 4), and Unspecified (n = 580).

- A horizontal arrow points right to: References removed (n = 1616). Reasons include Duplicates identified manually (n = 57), Duplicates identified by Covidence (n = 1559), Marked as ineligible by automation tools (n = 0), and Other reasons (n = 0).

2. Screening Phase:

- Studies screened (n = 2268). A horizontal arrow points right to Studies excluded (n = 2108).

- Studies sought for retrieval (n = 157). A horizontal arrow points right to Studies not retrieved (n = 0).

- Studies assessed for eligibility (n = 157). A horizontal arrow points right to Studies excluded (n = 110). Reasons for exclusion include: Not evaluating/assessing an intervention (n = 30), Not a fragile setting (n = 25), Systematic review (n = 16), Protocol for a study (n = 13), Not M H P S S intervention (n = 10), Participants have not directly or indirectly experienced G B V (n = 6), Realist review (n = 3), Not written in English (n = 2), Meta analysis (n = 1), Wrong outcomes (n = 1), scoping review (n = 1), Wrong study design (n = 1), and No results reported (n = 1).

3. Included Phase:

- Final box at the bottom: Studies included in review (n = 47).

Navigate back to Figure 1..

### Figure 2. Long description

A world map uses a color gradient to indicate the Number of Studies per country. A legend at the top center shows a gradient bar from light orange (1 study) to dark orange (10 studies). Most of the world is shaded light gray, indicating zero studies.

* In Africa, the Democratic Republic of the Congo has the highest concentration, shaded in dark orange. Surrounding countries including Ethiopia, Kenya, Uganda, Tanzania, and Zambia are shaded in medium orange. West African countries like Nigeria and Niger are shaded in light orange.

* In the Middle East and Asia, Pakistan is shaded in light orange, with smaller highlighted areas in Bangladesh and Malaysia.

* South Africa is also shaded in a very light orange tint.

Navigate back to Figure 2..
