# Supplementary material for: Mental health and psychosocial support interventions for individuals affected by gender-based violence in fragile contexts: A systematic review
Source: Glob Ment Health (Camb). 2026 Jul 22;13:e161. doi: 10.1017/gmh.2026.10272 (PMC13433338; doi:10.1017/gmh.2026.10272)
Supplement: Supplementary file 1 [file S2054425126102726sup001.docx]

**Supplementary File 1**

*Study Characteristics*

| **S. No.** | **Authors** | **Study Location** | **Study Design** | **Sample** | **Form of GBV** | **MHPSS Intervention** | **Outcomes** |
| --- | --- | --- | --- | --- | --- | --- | --- |
| 1. | Abdulah & Abdulla, 2019 | Sinjar, Northern Iraq | Pre-test post-test evaluation | n= 14  Age range: 10-27 years; Mean age= 19.21 years Kurdish Yezidi women who left their hometown following the ISIS invasion and who were then living in Sharya IDP Camp | Witnessed, experienced or escaped sexual GBV | Intensive non-invasive art-based intervention programme | Short-term mental well-being  Socio- psychological well-being |
| 2. | Asghar et al., 2018 | Khyber-Pakhtunkhwa province, Pakistan | Pre-test post-test design and qualitative in- depth interviews | n= 15 (qualitative interviews) n= 78 (pre-test post-test)  Mean age= 15.31 years | Risk of GBV (form not specified) | Creating Opportunities through Mentorship, Parental Involvement, and Safe Spaces (COMPASS) adolescent life skills intervention | Safety; Movement; Comfort discussing topics with caregivers; Gendered rites of passage; Social support networks; Perceptions of support for survivors of violence; Knowledge of support for survivors of violence |
| 3. | Bass et al., 2013 | South Kivu, Democratic Republic of Congo | Randomized controlled trial | n= 405  Mean age ( CPT)= 36.9 years; Mean age (individual support)= 33.8 years  Current and former female clients of psychosocial assistants, with clinically significant psychological problems | Experienced or witnessed sexual violence | Cognitive processing therapy | PTSD symptoms;  Combined depression and anxiety symptoms;  Functional impairment |
| 4. | Bass et al., 2016 | South Kivu, Democratic Republic of Congo | Randomized controlled trial | n= 301  Mean age= early 40s  Women with significant functioning problems or mental health symptoms, likely related to trauma | Survivors and witnesses of Sexual violence | Village Savings and Loans Associations (VSLA) | Economic outcomes; Social functioning; Mental health symptom and daily functioning |
| 5. | Bass et al., 2022 | Villages in the DRC | Longitudinal cohort study | n= 134  Mean age: 38.6 years  100% female Purposive sampling from participants of a parent study | Direct and in-direct experiences of sexual violence | Cognitive processing therapy | Depression, anxiety and PTSD symptoms; functional impairment |
| 6. | Bryant et al., 2017 | Nairobi, Kenya | Randomized controlled trial | n= 421  Mean age= 35 years  Women from every 10^th^ household in peri-urban slums | Prior or current history in GBV | Project manegement + | Psychological distress at 3 months post-intervention; WHODAS scores; PTSD scores; PSYCHLOPS scores; Exposure to life event scores; Differences in health care utilization between the PM+ group and control group. |
| 7. | Cao et al., 2020 | West Mamprusi and Nabdam Districts of Ghana | Secondary data analysis of a randomized controlled trial | n= 374  Mean age=  26.95  Women part of Community Pregnancy Surveillance Targeted Education Sessions (C-PrES) groups | Intimate partner violence | Integrated Mothers and Babies Course & Early Childhood Development (iMBC/ECD)- mental health intervention | IPV; Mediators: social support, couple communication |
| 8. | Chaudhury et al., 2016 | Southern Kayonza District, Rwanda | Randomized controlled trial; interviews | n= 295 (82 families)  Mean age= 41.03 years  Families affected by caregiver HIV | Intimate partner violence | Family Strengthening Intervention for HIV-affected families (FSI-HIV) | Problematic caregiver alcohol use; IPV; Family conflict; Child and family well-being |
| 9. | Cikuru et al., 2021 | South Kivu province, Democratic Republic of Congo | Mixed method: Pre-test post-test and qualitative interviews | n= 167  Age range: 15-69 years; Median age= 37 years  Women referred to psychology services to a rural hospital where the program was running | Conflict-related trauma and sexual violence | Healing in Harmony (HIH)- a form of music therapy | Depression, anxiety, and PTSD; Perceptions of life and self |
| 10. | Cohen, 2013 | Lago Agrio, Ecuador and a nearby Colombian refugee settlement. | Interviews | n= 28  Range: 17-60 years  Clients in a women’s shelter for survivors of GBV and residents from a refugee settlement | GBV in the context of armed conflict: intimate partner violence, rape, conflict related rape, survival sex, sex trafficking, childhood sexual abuse, and/or incest. | Common threads program: group-based textile making process plus psychosocial skill building therapeutic activities | Therapeutic benefits for participants in the Common Threads pilot project |
| 11. | Dawson et al., 2016 | Nairobi, Kenya | Randomized controlled trial | n= 70  Mean age (PM+)= 33; Mean age (control group)= 37  Women in every tenth household in the assessors’ allocated area | GBV (form not specified) | Problem Management Plus: an evidence-informed psychological intervention developed by the WHO | Feasibility; Psychological distress; Functioning; Gender-based violence; Post-traumatic stress symptoms |
| 12. | Falb et al., 2023 | North Kivu, Democratic Republic of Congo | Randomized controlled trial | n= 404  Mean age (women)= 32 years; Mean age (men)= 36 years  Couples registered for the Safe at Home program | Domestic violence | Safe at Home programme: a community-based discussion group series | Family functioning; IPV; harsh discipline |
| 13. | Ferrari et al., 2022 | Ghana, Kenya, Pakistan, Rwanda, South Africa, and Zambia | Trial-based analysis; multi-country cost-effectiveness analysis | N/A | Sexual and/or physical IPV (except Kenya and Pakistani interventions which focused on children) | 6 interventions across 6 countries- interventions targeted different impact mechanisms defined using theories of change: economic empowerment alongside a gender empowerment component (South Africa and Rwanda); psychological empowerment (Kenya and Pakistan), including self-defense (Kenya) and psychotherapeutic (Zambia); gender attitudes and behaviours (South Africa, Ghana, Pakistan, and Rwanda), and social norms change (Ghana and Rwanda) | Cost per DALY (disability adjusted life years) averted |
| 14. | Friedberg et al., 2023 | Nairobi, Kenya | Randomized controlled trial | n= 4091  Age range: 10-14 years  80% female, 20% male  Class 8 students students across 94 schools | Direct experience of rape | Empowerment and self defense programme | Depression, anxiety and PTSD; Prevalence of mental health conditions by history of sexual violence; Effect of empowerment intervention on mental health outcomes; Conditional average treatment effect (CATE) of the empowerment intervention on mental health outcomes |
| 15. | Greene et al., 2021 | Nyarugusu refugee camp in Tanzania | Randomized controlled trial | n= 311  Mean age= 33.5 years  Women’s group members in a refugee camp | Intimate partner violence | Nguvu: an integrated intervention comprised of CPT and advocacy counselling | Psychological distress; IPV; Functional impairment |
| 16. | Greene et al., 2022 | Nyarugusu refugee camp, Tanzania | Randomized control trial using a qualitative approach | n= 29  Nguvu participants, staff members, community advisory board members and humanitarian practitioners | Intimate partner violence | Nguvu intervention: cognitive processing therapy and advocacy counselling. | **Nguvu Participants:** Perceived and experiences associated with attending Nguvu activities, perceived impacts of the intervention, unexpected consequences and outcomes of participation in Nguvu, barriers and facilitators of attendance, and intervention acceptability.  **Facilitators and supervisors:** perceived impact of the intervention on participants, challenges associated with delivering the intervention, participant adherence and engagement, unexpected outcomes and consequences of implementing the Nguvu intervention, logistical challenges associated with scheduling and organizing intervention sessions, facilitator motivation, sufficiency of training and preparation, fidelity, and coordination |
| 17. | Hall et al., 2014 | South and North Kivu, Democratic Republic of Congo | Secondary data analysis of a randomized controlled trial | n= 405  Mean age (CPT group) 36.89 years  Mean age (control) = 33.77 years  Women from 14 villages in Eastern DRC | Sexual violence | Cognitive processing therapy | 4 social capital domains: contact with social network, group partipation, financial social support network and instrumental social support network; Structural social capital;  Improved emotional support seeking. |
| 18. | Heerden et al., 2020 | Maseru and Berea districts, Lesotho | Quasi-experimental study | n= 126  Range= 10-24 years  Adolescent girls and young women within community councils | Sexual GBV | Determined, Resilient, Empowered, AIDS-Free, Mentored, and Safe (DREAMS) Partnership | Reduction in risk behaviours |
| 19. | Hirani et al., 2010 | Karachi, Pakistan | Randomized controlled trial | n= 24  Range: (age of most women) 25-35 years  100% female | Intimate partner violence | Economic Skill Building (ESB) | Depression; Self-reports of abuse; Self-efficacy; Self-reported employment status |
| 20. | Ismayilova et al., 2018 | Nord Region, Burkina Faso | Randomized controlled trial | n= 360  Mean age= 37.21 years  Women from ultra poor households in villages | Intimate partner violence | Trickle up (economic strengthening intervention) and Trickle up plus (economic strengthening intervention with a gender sensitive family coaching component) | Women’s empowerment; Domestic violence; Quality of marital relationship; Decision-making power; Gender equality beliefs; Financial autonomy |
| 21. | James et al., 2021 | Syrian refugees in Lebanon and Rohingya in Malaysia | Study 1: Pre-test post-test design Study 2: randomized controlled trial in Malaysia, matched cluster comparison in Lebanon | Study 1  n= 148 Malaysia 35 women, 37 men  Mean age= 31 years Lebanon 41 women, 33 men Mean age= 34  Study 2:  n= 500  Malaysia 121 women, 119 men  Lebanon 131 women, 129 men | Intimate partner violence | Study 1: Three day workshops, utilizing a social norms-based mental health-integrated approach Study 2: poster campaigns | **Study 1:** Acceptability of violence; Ideas about gender relationships; Help-seeking for mental health needs, victim and perpetrators of IPV; Mental health (distress symptoms); Functional impairment; Self-efficacy; Community efficacy; Social cohesion;  Coping   **Study 2:** Acceptability of violence; Individual beliefs about gender relations; Relationship problem-solving efficacy; Help-seeking beliefs |
| 22. | Kalra, Habumugisha, & Shankar, 2024 | Kigrme camp, Nyamagabe, Rwanda | Randomized controlled trial | n= 1061  Women mean age = 33. 0 (SD= 6.4)  Partner Mean age = 39.7 (SD=9.18)  1061 females and 801 of which had male partners  Women in households in a refugee camp, who reported living with an intimate partner for at least the last six months | Intimate partner violence | Nimenye Mpinduke, Nigeire (NMN) training: an adapted version of a personnel agency training | Incidence of IPV;  Incidence of non-partner violence; Uptake and use of clean cooking system; Engagements in income-generating activities in the past 6 months; Engagement in skill learning; Depression and anxiety scores; PTSD scores; Food insecurity scores; Self-efficacy scores; Shift and persist scores; Ryff social agency scores |
| 23. | Kane et al., 2021 | Urban Zambia | Randomized controlled trial | n=190  97 women, 93 men  Couples in which the woman reported IPV | Intimate partner violence | Common elements treatment approach (CETA), | Physical/sexual violence and threatened violence against women; Alcohol use |
| 24. | Kim et al., 2021 | Siaya County, Kenya | Quasi-experimental longitudinal study | n= 417  Mean age= 26.4 years  Women recruited from existing community-based care groups who were pregnant or had a child under the age of two years | 40.7% of sample experienced Physical/Sexual Violence by Husband/Partner in the Past Year  22 % of sample experienced Emotional Violence by Husband/ Partner in the Past Year  44.9% of sample experienced Controlling Behavior by Husband/ Partner in the Past Year | Culturally adapted version of ‘Integrated Mothers and Babies Course and Early Childhood Development’ (iMBC/ECD) | Maternal depression; Children’s social and emotional development |
| 25. | Koegler et al., 2019 | South Kivu, Democratic Republic of Congo | Interviews | n= 12  Range: 18-60 years  Solidarity group members who were thriving and members who were struggling | conflict-related sexual violence (perpetrated by a combatant) | Solidarity groups | Mental and physical health  skills and other benefits gained from joining the group  contributions they themselves made to the group, their unmet needs |
| 26. | Koris et al., 2021 | Jordan and Niger | In-depth interviews (IDIs) and focus group discussions (FGDs), paired interviews (PIs) and participatory research activities (PARs) | n= 469  Families who had an adolescent girl aged 10-14 years, an older male sibling and a male and female caregiver | GBV (form not specified) | Sibling Support for Adolescents in Emergencies (SSAGE) | Impact of SSAGE on male-female sibling dyad |
| 27. | Latif et al., 2020 | Karachi, Pakistan | Randomized controlled trial and pre–post evaluation | n= 50  Mean age= 27.4  100% female | Domestic violence | Culturally adapted trauma-focused CBT-based guided self-help program(CatCBT GSH) | Feasibility; Acceptability; Scores on Impact of Event Scale-Revised (IES-R), Hospital Anxiety and Depression Scale (HADS), WHO Disability Assessment Schedule 2 (WHO DAS 2) |
| 28. | Lilleston et al., 2018 | Wadi Khaled, Lebanon | Qualitative interviews | *n* (adolescent girls) = 26; *n* (Syrian refugee women) = 38; *n* (staff) = 11  Mean age= 24 years  Range= 14-50 years  Syrian refugees living in tented settlements (ITS), or houses and shelters in host communities | GBV (form not specified) | GBV mobile service delivery approach: psychosocial support activities, risk mitigation activities, individual case management | the extent to which the intervention was able to meet (1) international standards to guarantee the safety of GBV survivors and quality of services and (2) the support needs of refugee women and girls. |
| 29. | Mannella, Ahmadb, & Ahmada, 2018 | Afghanistan | Semi-structured interviews | n (survivors of GBV)= 20; n (staff)= 8  Women residing in safe houses following violence from their husbands or other male authority figures | Violence from husbands or other male authority figures | Narrative storytelling | Potential for storytelling to support women's mental health |
| 30. | Meffert et al., 2021 | Kisumu, Kenya | Randomized controlled trial | n= 256  Mean age= 37 years  HIV–positive women receiving FACES-Kisumu HIV care in an HIV clinic | GBV (form not specified) | Interpersonal psychotherapy | MDD and PTSD at 3 months follow-up; symptom measures of depression and PTSD, intimate partner violence (IPV), and disability |
| 31. | Mogga, 2017 | North Uganda | Not specified | Not specified | Sexual GBV | Cognitive behavioural treatment therapy for trauma (CBTT) | Not specified |
| 32. | Murray et al., 2018 | South and North Kivu, Democratic Republic of Congo | Secondary data analysis of a randomized controlled trial | n= 405  Mean age (CPT group)= 37  Mean age (control)= 34  Women from 16 villages in DRC | Witnessed or experienced sexual violence | Cognitive processing therapy (CPT) | Felt stigma;  Depression and anxiety; Functional impairment; Posttraumatic stress |
| 33. | Murray et al., 2020 | Lusaka, Zambia | Randomized controlled trial | n= 496; 248 couples  50% men, 50% women  Women and men who were married, dating, or in a relationship | Intimate partner violence | Common Elements Treatment Approach (CETA) | Women’s experience of IPV ; Male partner’s hazardous alcohol use |
| 34. | Murray et al., 2021 | Lusaka, Zambia | Semi-structured interviews; focus groups | n= 44  23 women, 21 men  Couples randomized to CETA in the RCT assessing the program’s effectiveness for reducing IPV and alcohol use the original study | Intimate partner violence | Common Elements Treatment Approach (CETA) | Mechanisms of behaviour change related to male IPV perpetration and women’s ability to negotiate safety and avoid potential violence. |
| 35. | Mutisya, Ngure, & Mwachar, 2018 | Kisumu County, Kenya | Quasi-experimental study | n= 288  Range:  18-45 years  Pregnant women subjected to GBV, seeking antenatal care services in health facilities | Physical, sexual, psychological and economic violence by intimate partners, family members or any other persons | Secondary prevention approach for early detection and reduction or elimination of GBV during pregnancy | GBV; Antepartum depression;  Economic violence, Physical and sexual violence by non-intimate partners; Perpetration of violence against an intimate partner |
| 36. | Namya et al., 2022 | Kampala, Uganda | Pre-test post-test evaluation | n= 11  Mean age= 17; Range= 15-20 years  Current female adolescent clients residing in a Willow shelter (shelter for victims of human trafficking) | Human trafficking | Healing and Resilience after Trauma (HaRT) Yoga | Depression; Self-rated emotional health; Self-rated physical health |
| 37. | Naved et al., 2024 | Rangpur, Bangladesh | Randomized controlled trial | n (girls)= 1275  Range= 12-16 years; Mean age = 13.50  Never-married girls who were residents in the villages included in the study  n (community members)= 626 | Child marriage | Tipping Point Initiative (TPI): intervention to empower girls and address social norms related to child marriage | Hazard of girl child marriage; Girls’ self-efficacy; Collective efficacy; Girls’ knowledge regarding Sexual and Reproductive Health and Rights (SRHR); Girls’ positive attitudes regarding gender roles; Girls’ endorsement of control of adolescent girls by family members; Girls’ endorsement of justification of girl-beating; Girls’ cohesion; Girls’ confidence in negotiation skills; Girls’ mobility; Girls’ participation in financial activities; Girls’ connectedness with parents; Community positive social norm (community members’ reporting) |
| 38. | O’Callaghan et al., 2013 | North Kivu, Democratic Republic of the Congo | Randomized controlled trial | n= 52  Age range: 12-17 years  Girls who were rescued from brothels by a local NGO | Direct and indirect experience of rape or sexual abuse. | Trauma-focused  cognitive behavioural therapy (TF-CBT) | PTSD symptom scores; depression, anxiety, conduct and prosocial behaviours |
| 39. | Park, 2022 | Monrovia, Liberia | Randomized controlled trial | n= 395  Mean age= 29 years  Women currently partnered or who have had an intimate partner 12 months prior to the survey | Intimate partner violence | Women Training and Integration (WIN) Program | Prevalence of IPV; Psychological well-being; Economic livelihoods; Social norms related to IPV acceptability |
| 40. | Puffer et al., 2020 | Eldoret, Kenya | Pre-test post-test evaluation | n= 10 families  Mean age (adolescents)= 14 years, Range= 12-17; Mean age (caregiver)= 39 years, Range= 26-61  Families who counsellors in the community believed exhibited persistent patterns of dysfunctional family interactions | Intimate partner violence | Tuko Pamoja (TP), “We are together” in Kiswahili: a family therapy intervention | Family functioning; Mental health; Harsh discipline; Couples’ violence and harshness; Alcohol use and alcohol- related conflict |
| 41. | Robjant et al., 2019 | Eastern Democratic Republic of Congo | Randomized controlled trial | n= 92  Range= 16-25 years  Female former child soldiers in DRC. | Sexual and gender based violence | Narrative Exposure for Forensic Offender Rehabilitation (FORNET) | PTSD; Appetitive aggression; Depression; Social variables; Current violent behaviour; Feelings of guilt; Increasing social acknowledgement |
| 42. | Sarnquist et al., 2014 | Huruma/Mathare, Dandora, Kibera, and Mukuru Kwa Reuben informal settlements of Nairobi | Randomized controlled trial | n= 2406  Range= 13- 20 years  Adolescent girls attending one of 31 secondary schools selected | Sexual assault | Empowerment and Self-Defence Training | Reduction in the incidence of sexual assault |
| 43. | Shair, Akhter, & Shama, 2019 | Cox’s Bazaar, Bangladesh | Case study | n= 2  Age: 21 years and 30 years Rohingya refugee women | GBV, rape, sexual assault | Inclusion of psychosocial support in GBV interventions | Perception of how integration of the PSS approaches into GBV programs contributed to well-being as an individual and community. |
| 44. | Siu et al., 2024 | Uganda | Quasi-experimental pre-post design | n (parents)=484  n( children)= 212  Mean age (children)= 11.7 Mean age (parents)= 38.9  269 mothers, 215 fathers; 117 girls, 95 boys  Parents with children aged 0-17 years could enrol in the intervention | Intimate partner violence | Parenting for Respectability Programme (PfR): addresses familial processes associated with poor parenting and intimate partner violence | Harsh parenting; Dysfunctional partner relationships (especially IPV); Positive parenting ; Respectful child behaviour; Carer-child conflict; Parent self-inefficacy;  Provision of necessities as proxy for child neglect; Co-parenting; Partner involvement in caregiving; Community/collective parenting; Attitudes towards gender socialization; Knowledge of child development |
| 45. | Stark et al., 2018 | Benishangul-Gumuz regional state of Ethiopia | Randomized controlled trial | n= 919  Mean age= 14 years  Adolescent girls in refugee camps | Interpersonal violence- sexual, physical, emotional violence, transactional sex, child marriage | Creating Opportunities through Mentorship, Parental Involvement, and Safe Spaces (COMPASS) adolescent life skills and safe spaces programme | Sexual violence in the previous 12 months; exposure to physical violence, emotional violence, transactional sex, child marriage and feelings of safety |
| 46. | Thabet et al., 2019 | Gaza, Palestine | Pre-test post-test evaluation | n=110  Range: 16-42 years  Mean age=25.6  Women receiving services for domestic violence-related concerns | Domestic violence | Community intervention of group counselling and vocational training | Domestic violence scores  Mental health scores  Resilience scores |
| 47. | Veale et al., 2020 | Lebanon | Interviews; focus groups; observation | n (in men's focus groups) = 130 n (in women’s focus groups)= 28 n (adolescent children)= 11  Syrian refugees in Lebanon | GBV (form not specified), including sexual violence | Engaging Men to promote resilient communities | Gender-based violence; sexual harassment and exploitation; early marriage and child labour. |
