# Supplementary material for: Mental health and psychosocial support interventions for individuals affected by gender-based violence in fragile contexts: A systematic review
Source: Glob Ment Health (Camb). 2026 Jul 22;13:e161. doi: 10.1017/gmh.2026.10272 (PMC13433338; doi:10.1017/gmh.2026.10272)
Supplement: Peer review history

# Reviewer report: Review: Mental health and psychosocial support interventions for individuals affected by gender-based violence in fragile contexts: A systematic review — R0/PR2

**DOI:** 10.1017/gmh.2026.10272.pr2

This narrative review clearly documents the paucity of studies on efficacy of GBV interventions to address mental health and social support. A strength of the work is its broad description of the extant literature and how it highlights the range of intervention approaches and the wide variation in geography, sampling strategies and source populations, intervention level, and type of practitioners. Notably, among the 47 studies identified, four types of outcomes were --mental health outcomes (n=24), incidences of GBV (n=18), social- and community-level (n=7), and women’s economic empowerment outcomes (n=4).The most evidence is available for mental health outcomes, with several studies employing similar approaches and similar measures enabling assessment of replicability and robustness of the findings.

However, a major concern of this narrative review is the drawing of substantive conclusions based on limited evidence either because there are only a few studies for a given outcome or because a given intervention strategy has not been replicated. Emphasize robust findings that have been replicated across studies (eg CBT). Emphasize preliminary nature of findings based on only a few or single studies. Care should be taken throughout the manuscript to ensure that conclusions are not based on single studies (eg conclusions about efficacy of yoga, art page 21, bottom). Perhaps preliminary evidence suggests that these may be useful, but lack of replication is a concern. Given there are only 7 community intervention studies, not all of which were economic, conclusions bottom of 21 top of 22 may be overstated.

Paragraphs on pages 22 and 23, make important points but do not seem to develop from the evidence presented, or cross-study findings from the narrative review (again take care in broad generalizations based on few studies). Either tie these paragraphs into the results or clearly identify as additional considerations for future intervention studies.

In addition please consider the following specific points.

Abstract

1. Please correct “… literature were searched for studies published between 21st June and 24th July 2024.” According to your methods section this time window is the time window in which the search was conducted. You state there were no time limits set on the date of publication.

Methods

1. Please provide a definition of what you include in “MHPSS”

2. Please list or provide a table of the key words searched.

Results

1. Page 12 paragraph under “Level 3: Location and Setting of Interventions” you enumerate settings for 30 of the 47 studies, what settings are used in the other 17 studies. If not stated then please say so.

2. Page 13, similarly you list sampling strategy for 42 of the 47 studies, what sampling strategy did the other 5 studies use?

3. Page 13, Number of studies in paragraph starting on line 212 also don’t add to 47

4. Very confused by enumeration of levels that go back and forth between levels in this section

5. Page 16, last sentence. Why do the Friedberg and Park references not find an effect on mental health. What is different about their population, intervention, measurement, study power, etc that might explain this?

Discussion

1. Page 22 lines 426-431 see also

Alexandre, A.B., Kasherwa, A., Balegamire, J.B. et al. Bouncing back after trauma: music therapy, gender, and mental health in conflict-ridden settings. Discov Ment Health 5, 15 (2025). https://doi.org/10.1007/s44192-025-00137-1

2. Page 24 lines 454-458: Was self-esteem assessed in these studies? I don’t see it discussed in results summaries. If not state it as a gap in knowledge and more strongly justify inclusion of this issue in the discussion.

3. Lines 459-466 – do the studies you review support an increase in GBV following economic interventions? (not discussed), in addition to presenting the potential problem (and whether there are studies documenting it) please contextualize the concern based on approaches used and findings in the studies you review. As a number of studies reviewed do incorporate men.

4. Paragraphs 459 and 467, the UN R.E.S.P.E.C.T strategy for reducing GBV against women speaks specifically to improving relationships (the R) and inclusion of men in strategies. Some reference to this global model and associated intervention studies is warranted.

See https://www.unwomen.org/en/digital-library/publications/2015/11/prevention-framework

and https://www.unwomen.org/en/digital-library/publications/2020/07/respect-women-implementation-package

5. Please include a discussion about the lack of comparative efficacy studies (many interventions may help but what interventions have the strongest impacts?).

# Reviewer report: Review: Mental health and psychosocial support interventions for individuals affected by gender-based violence in fragile contexts: A systematic review — R0/PR3

**DOI:** 10.1017/gmh.2026.10272.pr3

The authors have provided a well-written article that contributes to our understanding the impact of MHPSS interventions on key outcomes of mental health, IPV, economic and social norms in conflict/fragile settings. I have few suggestions for improving the article. 1. PM+ is problem management, not project management. 2. It is unclear to me why the authors included the qualitative studies, as they were not well integrated into answering the three questions proposed related to intervention type, outcomes and efficacy. I would consider focusing the manuscript on the quantitative studies, as they best answer the authors study questions and are the focus of the findings and discussion. I appreciate the opportunity to review the manuscript and think it will contribute to the knowledge on mental health and psychological support programs in humanitarian settings.

# Reviewer report: Review: Mental health and psychosocial support interventions for individuals affected by gender-based violence in fragile contexts: A systematic review — R0/PR4

**DOI:** 10.1017/gmh.2026.10272.pr4

This manuscript presents well-executed systematic review of mental health and psychosocial support interventions for individuals affected by gender-based violence in fragile and humanitarian contexts.

A few points of clarification or further elaboration would improve the paper.

1. There is no clear definition of “fragile contexts”. It would be helpful to specify whether these contexts exclusively refer to humanitarian settings, internally displaced populations, or a broader set of fragile or conflict-affected environments, and to explain how this classification was operationalized during study selection.

2. Could authors provide a bit more background in the main text describing the range and nature of the outcomes assessed across included studies. Specifically, further detail on the rates of mental health and GBV incidence, as well as a clearer description of social- and community-level outcomes and women’s economic empowerment outcomes would improve interpretability.

3. With respect to methodology, can the authors specify if the 20% of titles and the 20% of full text reviewed were the same papers or was the randomized selection done independently – that is it would be useful to clarify whether these were the same 20% of studies or two separate random samples.

4. The review includes a wide range of populations (e.g., women, adolescents, couples, perpetrators, and communities), intervention types (including CBT-based approaches, alternative therapies, economic empowerment interventions, and social norms programming), and outcomes (mental health, GBV incidence, and economic indicators). While this inclusivity is a strength, it also introduces substantial heterogeneity. In much of the discussion, I found that it tended to read as a descriptive summary. The analysis and discussion could benefit from more critical comparison across intervention characteristics (such as intervention intensity, duration, delivery agents, and gender inclusion) and this would enhance analytical depth.

5. Did the authors find significant difference in measurements in outcomes across studies? It would the authors pose any critique on their cultural validity, reliability, or consistency across diverse humanitarian settings. Related to the prior comment - it may be important to note that substantial heterogeneity likely exists across settings, including differences between acute and protracted crises and variation in baseline prevalence of key outcomes.

6. In the limitation section or discussion, it should be noted the evidence base is overwhelmingly focused on women and girls. Interventions involving men, boys, or gender-diverse individuals are sparse and under-analyzed (if there are any).

7. I would add a note in the limitations section briefly discussing the potential for publication bias and the implications of excluding non-English language studies.

8. One further note – regarding the study by Kalra and colleagues (2024), the manuscript notes that the intervention was associated with higher rates of probable anxiety, depression, and PTSD in the intervention group compared to controls. It is important to contextualize this finding by noting that overall rates of mental health disorders in the protracted settlement were low (approximately 5%). While the authors suggest a potential explanation, acknowledging that other contextual or methodological factors may have contributed to this result would provide a more balanced interpretation.

Overall, the synthesis is comprehensive, but the analyses could be strengthened by moving beyond descriptive summaries toward more critical comparison across intervention types and settings.

# Editor report: Recommendation: Mental health and psychosocial support interventions for individuals affected by gender-based violence in fragile contexts: A systematic review — R0/PR5

**DOI:** 10.1017/gmh.2026.10272.pr5

**Affiliations:** Global Health Section, University of Copenhagen, Denmark

Dear authors,

Thank you for submitting your work to our journal. We have now received evaluations from peer reviewers. Based on their evaluation and my own, we would like to invite you to make major revisions to your paper.

Sincerely,

Wietse Tol

# Editor report: Decision: Mental health and psychosocial support interventions for individuals affected by gender-based violence in fragile contexts: A systematic review — R0/PR6

**DOI:** 10.1017/gmh.2026.10272.pr6

**Affiliations:** London School of Hygiene & Tropical Medicine, United Kingdom of Great Britain and Northern Ireland

No accompanying comment.

# Reviewer report: Review: Mental health and psychosocial support interventions for individuals affected by gender-based violence in fragile contexts: A systematic review — R1/PR8

**DOI:** 10.1017/gmh.2026.10272.pr8

The authors have addressed my prior concerns. The manuscript is well-written, provides descriptive information requested on all studies, and appropriately highlights which findings are supported by more than one study.

Page 31 line 622

Word missing in this phrase (CAPS ADDED) “...where there was a comparator group, in almost all cases THE COMPARATOR WAS a control or waitlist condition, rather than a comparison of different active interventions against one another.”

# Reviewer report: Review: Mental health and psychosocial support interventions for individuals affected by gender-based violence in fragile contexts: A systematic review — R1/PR9

**DOI:** 10.1017/gmh.2026.10272.pr9

The authors address an important issue in fragile and conflict settings, related to defining types of MHPSS interventions implemented in fragile/conflict settings, what outcomes are targeted and measured and the efficacy of these interventions. The third question of efficacy, as I understand efficacy was not appropriate/or relevant to several of the interventions reviewed. Efficacy asks: Does this intervention work under ideal, controlled conditions? It’s tested in randomized controlled trials with tightly defined populations, strict protocols, highly trained delivery staff, and close monitoring. The goal is to establish that the intervention can produce an effect, essentially ruling out chance and isolating the active ingredients. Like a proof of concept under optimal circumstances. This is not the context or reality for most studies reviewed, the interventions reviewed were implemented in humanitarian settings, both in camps and outside camps, not highly controlled settings. Further, there were no/limited efficacy data presented in the result, especially how are the authors defining efficacy with the qualitative studies? I wonder if the focus was on effectiveness, does this MHPSS intervention work in the real world? It’s tested under routine practice conditions, more diverse and less selected populations, real-world delivery settings, community health workers rather than specialists, levels of adherence and fidelity? An intervention can be highly efficacious in a trial but fail to show effectiveness when rolled out, because real-world conditions introduce variation, and implementation challenges that controlled settings manage and filter out.

The discussion section should focus on answering each of the three study research questions/aims, specifically based on the review results. The writing is challenging to follow, for example there is a focus on increased male participation in MHPSS intervention, unclear which question (type, outcomes, efficacy) that this recommendation is responding to?

The manuscript has potential, but clarity is needed in the questions being asked, the results and the implications/recommendations based on the findings to the research questions.

# Editor report: Recommendation: Mental health and psychosocial support interventions for individuals affected by gender-based violence in fragile contexts: A systematic review — R1/PR10

**DOI:** 10.1017/gmh.2026.10272.pr10

**Affiliations:** Global Health Section, University of Copenhagen, Denmark

Dear authors,

Thank you for submitting a revised version of your manuscript. As you can see from the comments from the reviewers, there are still some suggestions to improve the manuscript. We are looking forward to receive a revised version of the manuscript.

# Editor report: Decision: Mental health and psychosocial support interventions for individuals affected by gender-based violence in fragile contexts: A systematic review — R1/PR11

**DOI:** 10.1017/gmh.2026.10272.pr11

**Affiliations:** London School of Hygiene & Tropical Medicine, United Kingdom of Great Britain and Northern Ireland

No accompanying comment.

# Editor report: Recommendation: Mental health and psychosocial support interventions for individuals affected by gender-based violence in fragile contexts: A systematic review — R2/PR13

**DOI:** 10.1017/gmh.2026.10272.pr13

**Affiliations:** Global Health Section, University of Copenhagen, Denmark

Dear authors,

Thank you very much for the further revision. We are pleased to accept your manuscript.

Sincerely, Wietse

# Editor report: Decision: Mental health and psychosocial support interventions for individuals affected by gender-based violence in fragile contexts: A systematic review — R2/PR14

**DOI:** 10.1017/gmh.2026.10272.pr14

**Affiliations:** London School of Hygiene & Tropical Medicine, United Kingdom of Great Britain and Northern Ireland

No accompanying comment.
